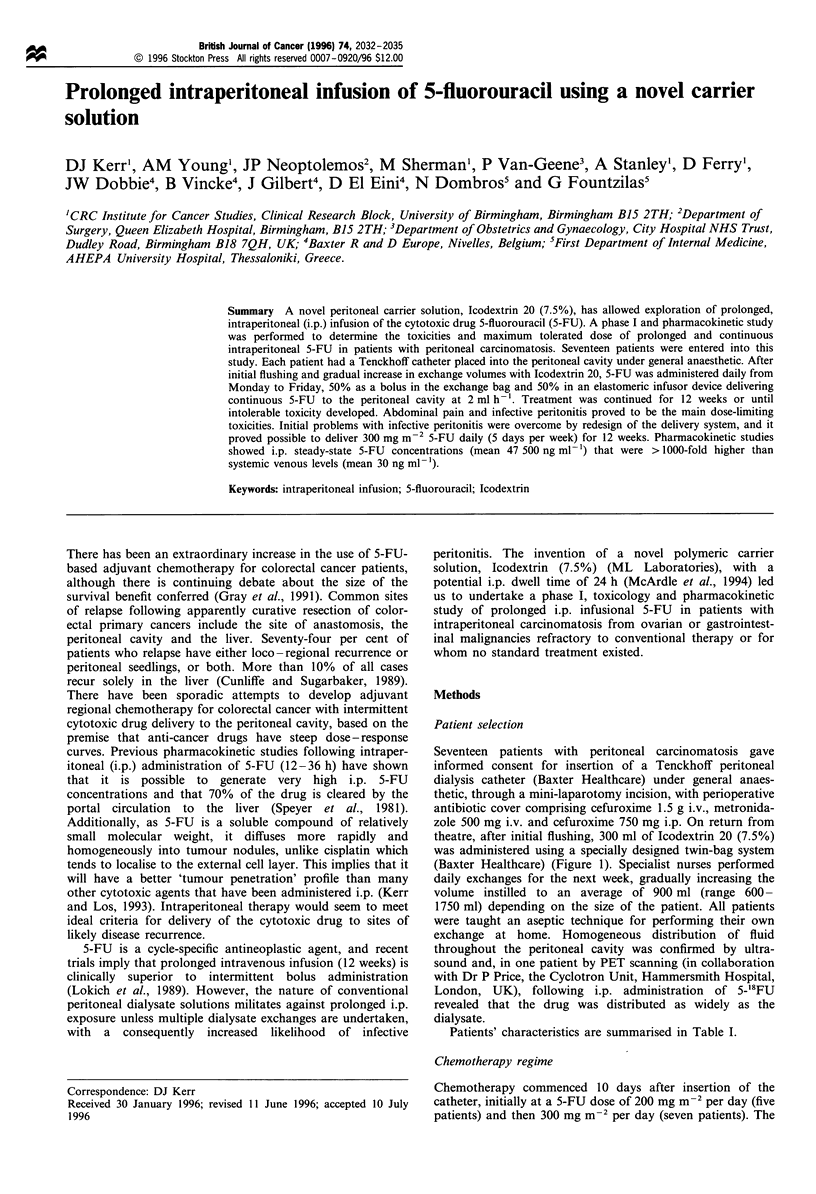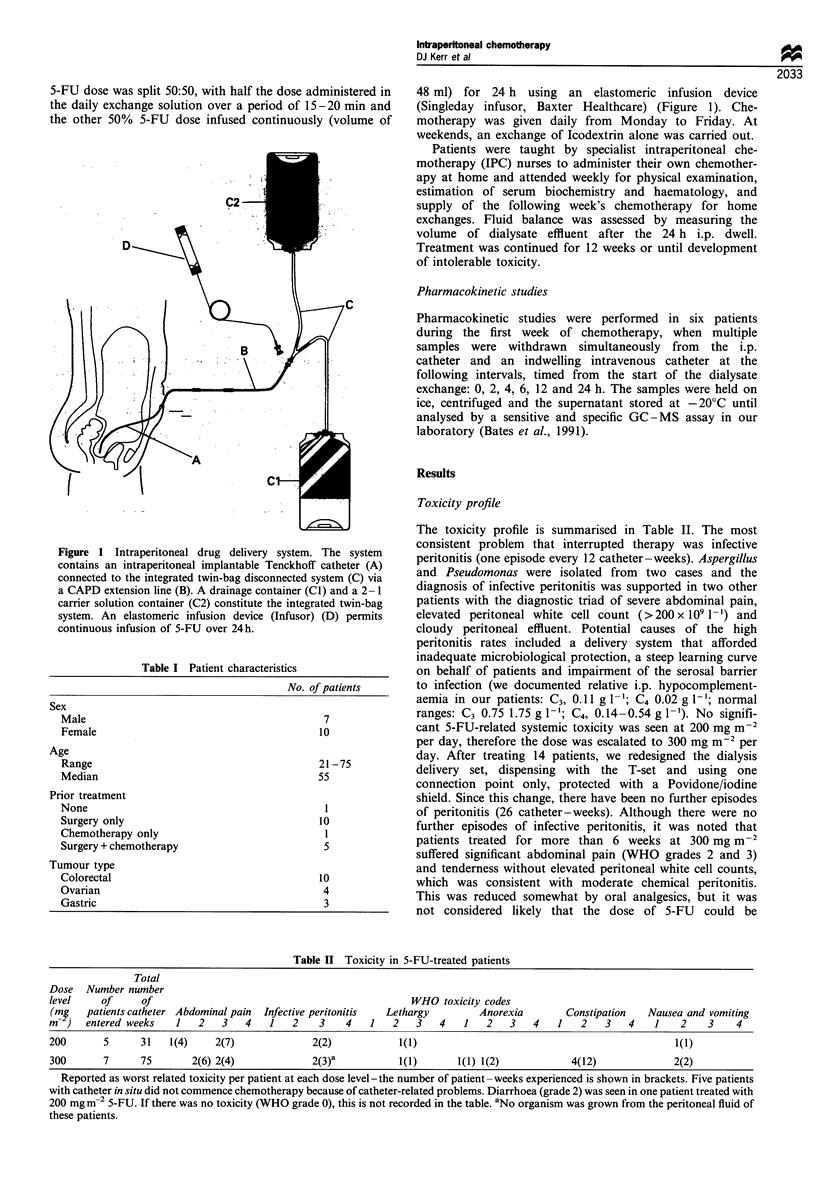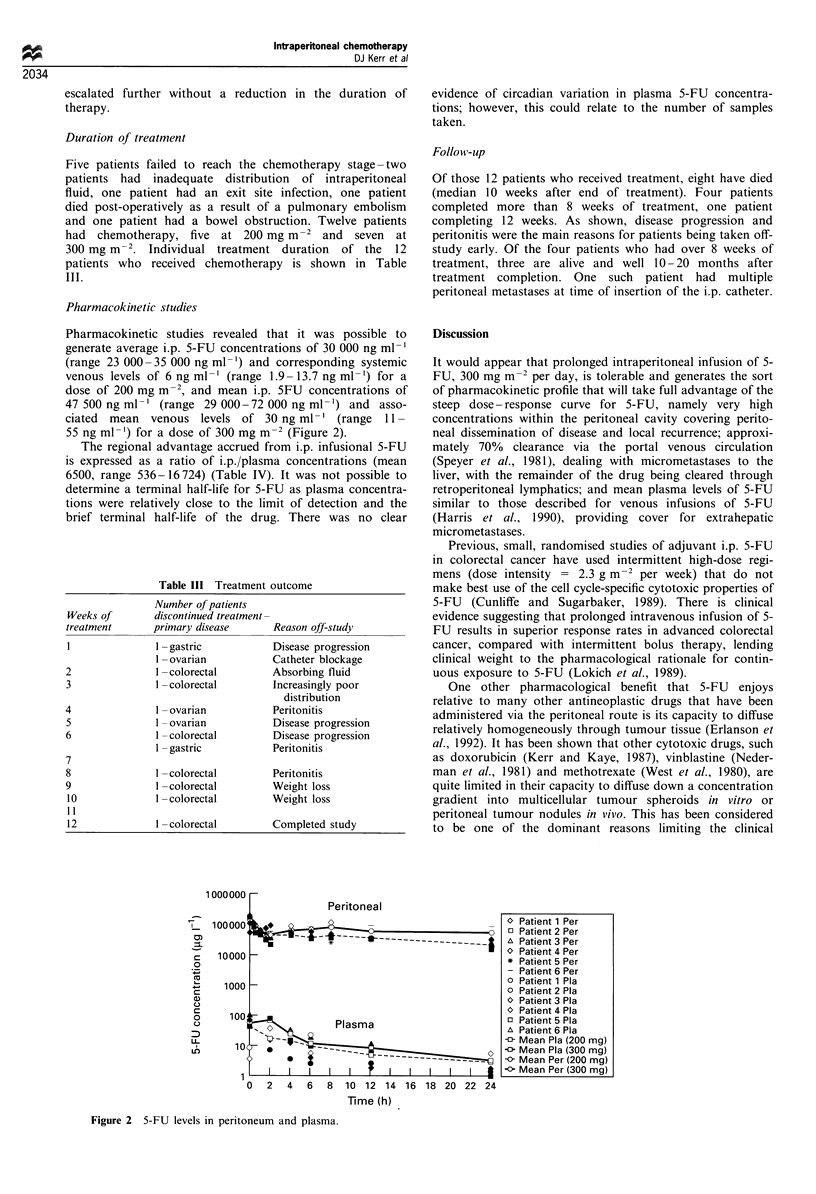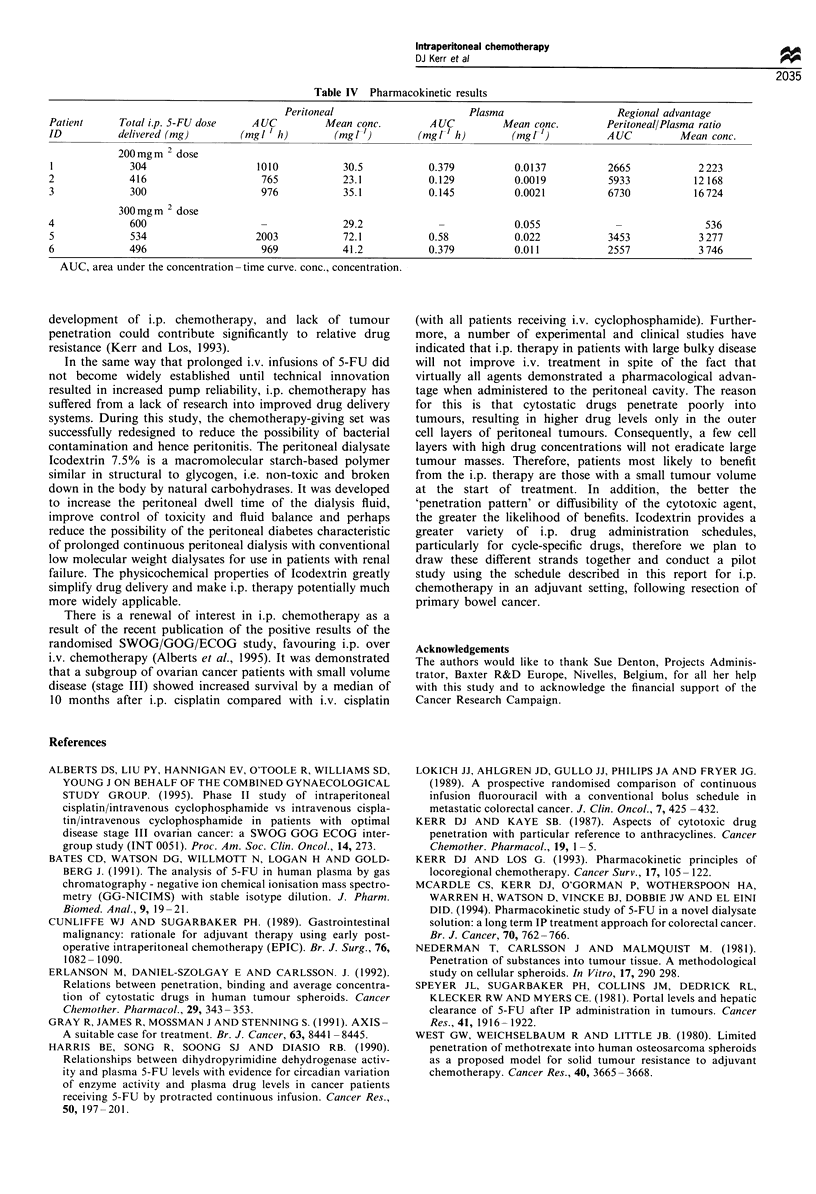# Prolonged intraperitoneal infusion of 5-fluorouracil using a novel carrier solution.

**DOI:** 10.1038/bjc.1996.672

**Published:** 1996-12

**Authors:** D. J. Kerr, A. M. Young, J. P. Neoptolemos, M. Sherman, P. Van-Geene, A. Stanley, D. Ferry, J. W. Dobbie, B. Vincke, J. Gilbert, D. el Eini, N. Dombros, G. Fountzilas

**Affiliations:** CRC Institute for Cancer Studies, University of Birmingham, UK.

## Abstract

A novel peritoneal carrier solution, Icodextrin 20 (7.5%), has allowed exploration of prolonged, intraperitoneal (i.p.) infusion of the cytotoxic drug 5-fluorouracil (5-FU). A phase I and pharmacokinetic study was performed to determine the toxicities and maximum tolerated dose of prolonged and continuous intraperitoneal 5-FU in patients with peritoneal carcinomatosis. Seventeen patients were entered into this study. Each patient had a Tenckhoff catheter placed into the peritoneal cavity under general anaesthetic. After initial flushing and gradual increase in exchange volumes with Icodextrin 20, 5-FU was administered daily from Monday to Friday, 50% as a bolus in the exchange bag and 50% in an elastomeric infusor device delivering continuous 5-FU to the peritoneal cavity at 2 ml h-1. Treatment was continued for 12 weeks or until intolerable toxicity developed. Abdominal pain and infective peritonitis proved to be the main dose-limiting toxicities. Initial problems with infective peritonitis were overcome by redesign of the delivery system, and it proved possible to deliver 300 mg m-2 5-FU daily (5 days per week) for 12 weeks. Pharmacokinetic studies showed i.p. steady-state 5-FU concentrations (mean 47 500 ng ml-1) that were > 1000-fold higher than systemic venous levels (mean 30 ng ml-1).


British Journal of Cancer (1996) 74, 2032-2035
© 1996 Stockton Press All rights reserved 0007-0920/96 $12.00

### Prolonged intraperitoneal infusion of 5-fluorouracil using a novel carrier solution

DJ Kerr[1], AM Young[1], JP Neoptolemos[2], M Sherman[1], P Van-Geene[3], A Stanley[1], D Ferry[1], JW Dobbie[4], B Vincke[4], J Gilbert[4], D El Eini[4], N Dombros[5] and G Fountzilas[5]

[1]*CRC Institute for Cancer Studies, Clinical Research Block, University of Birmingham, Birmingham B15 2TH;* [2]*Department of Surgery, Queen Elizabeth Hospital, Birmingham, B15 2TH;* [3]*Department of Obstetrics and Gynaecology, City Hospital NHS Trust, Dudley Road, Birmingham B18 7QH, UK;* [4]*Baxter R and D Europe, Nivelles, Belgium;* [5]*First Department of Internal Medicine, AHEPA University Hospital, Thessaloniki, Greece.*

**Summary** A novel peritoneal carrier solution, Icodextrin 20 (7.5%), has allowed exploration of prolonged, intraperitoneal (i.p.) infusion of the cytotoxic drug 5-fluorouracil (5-FU). A phase I and pharmacokinetic study was performed to determine the toxicities and maximum tolerated dose of prolonged and continuous intraperitoneal 5-FU in patients with peritoneal carcinomatosis. Seventeen patients were entered into this study. Each patient had a Tenckhoff catheter placed into the peritoneal cavity under general anaesthetic. After initial flushing and gradual increase in exchange volumes with Icodextrin 20, 5-FU was administered daily from Monday to Friday, 50% as a bolus in the exchange bag and 50% in an elastomeric infusor device delivering continuous 5-FU to the peritoneal cavity at 2 ml $h^{-1}$. Treatment was continued for 12 weeks or until intolerable toxicity developed. Abdominal pain and infective peritonitis proved to be the main dose-limiting toxicities. Initial problems with infective peritonitis were overcome by redesign of the delivery system, and it proved possible to deliver 300 mg $m^{-2}$ 5-FU daily (5 days per week) for 12 weeks. Pharmacokinetic studies showed i.p. steady-state 5-FU concentrations (mean 47 500 ng $ml^{-1}$) that were >1000-fold higher than systemic venous levels (mean 30 ng $ml^{-1}$).

**Keywords:** intraperitoneal infusion; 5-fluorouracil; Icodextrin

There has been an extraordinary increase in the use of 5-FU-based adjuvant chemotherapy for colorectal cancer patients, although there is continuing debate about the size of the survival benefit conferred (Gray *et al.*, 1991). Common sites of relapse following apparently curative resection of colorectal primary cancers include the site of anastomosis, the peritoneal cavity and the liver. Seventy-four per cent of patients who relapse have either loco-regional recurrence or peritoneal seedlings, or both. More than 10% of all cases recur solely in the liver (Cunliffe and Sugarbaker, 1989). There have been sporadic attempts to develop adjuvant regional chemotherapy for colorectal cancer with intermittent cytotoxic drug delivery to the peritoneal cavity, based on the premise that anti-cancer drugs have steep dose-response curves. Previous pharmacokinetic studies following intraperitoneal (i.p.) administration of 5-FU (12-36 h) have shown that it is possible to generate very high i.p. 5-FU concentrations and that 70% of the drug is cleared by the portal circulation to the liver (Speyer *et al.*, 1981). Additionally, as 5-FU is a soluble compound of relatively small molecular weight, it diffuses more rapidly and homogeneously into tumour nodules, unlike cisplatin which tends to localise to the external cell layer. This implies that it will have a better 'tumour penetration' profile than many other cytotoxic agents that have been administered i.p. (Kerr and Los, 1993). Intraperitoneal therapy would seem to meet ideal criteria for delivery of the cytotoxic drug to sites of likely disease recurrence.

5-FU is a cycle-specific antineoplastic agent, and recent trials imply that prolonged intravenous infusion (12 weeks) is clinically superior to intermittent bolus administration (Lokich *et al.*, 1989). However, the nature of conventional peritoneal dialysate solutions militates against prolonged i.p. exposure unless multiple dialysate exchanges are undertaken, with a consequently increased likelihood of infective peritonitis. The invention of a novel polymeric carrier solution, Icodextrin (7.5%) (ML Laboratories), with a potential i.p. dwell time of 24 h (McArdle *et al.*, 1994) led us to undertake a phase I, toxicology and pharmacokinetic study of prolonged i.p. infusional 5-FU in patients with intraperitoneal carcinomatosis from ovarian or gastrointestinal malignancies refractory to conventional therapy or for whom no standard treatment existed.

### Methods

### *Patient selection*

Seventeen patients with peritoneal carcinomatosis gave informed consent for insertion of a Tenckhoff peritoneal dialysis catheter (Baxter Healthcare) under general anaesthetic, through a mini-laparotomy incision, with perioperative antibiotic cover comprising cefuroxime 1.5 g i.v., metronidazole 500 mg i.v. and cefuroxime 750 mg i.p. On return from theatre, after initial flushing, 300 ml of Icodextrin 20 (7.5%) was administered using a specially designed twin-bag system (Baxter Healthcare) (Figure 1). Specialist nurses performed daily exchanges for the next week, gradually increasing the volume instilled to an average of 900 ml (range 600-1750 ml) depending on the size of the patient. All patients were taught an aseptic technique for performing their own exchange at home. Homogeneous distribution of fluid throughout the peritoneal cavity was confirmed by ultrasound and, in one patient by PET scanning (in collaboration with Dr P Price, the Cyclotron Unit, Hammersmith Hospital, London, UK), following i.p. administration of 5-$^{18}$FU revealed that the drug was distributed as widely as the dialysate.

Patients' characteristics are summarised in Table I.

### *Chemotherapy regime*

Chemotherapy commenced 10 days after insertion of the catheter, initially at a 5-FU dose of 200 mg $m^{-2}$ per day (five patients) and then 300 mg $m^{-2}$ per day (seven patients). The

Correspondence: DJ Kerr
Received 30 January 1996; revised 11 June 1996; accepted 10 July 1996

5-FU dose was split 50:50, with half the dose administered in the daily exchange solution over a period of 15–20 min and the other 50% 5-FU dose infused continuously (volume of 48 ml) for 24 h using an elastomeric infusion device (Singleday infusor, Baxter Healthcare) (Figure 1). Chemotherapy was given daily from Monday to Friday. At weekends, an exchange of Icodextrin alone was carried out.

Patients were taught by specialist intraperitoneal chemotherapy (IPC) nurses to administer their own chemotherapy at home and attended weekly for physical examination, estimation of serum biochemistry and haematology, and supply of the following week's chemotherapy for home exchanges. Fluid balance was assessed by measuring the volume of dialysate effluent after the 24 h i.p. dwell. Treatment was continued for 12 weeks or until development of intolerable toxicity.

**Figure 1** Intraperitoneal drug delivery system. The system contains an intraperitoneal implantable Tenckhoff catheter (A) connected to the integrated twin-bag disconnected system (C) via a CAPD extension line (B). A drainage container (C1) and a 2-1 carrier solution container (C2) constitute the integrated twin-bag system. An elastomeric infusion device (Infusor) (D) permits continuous infusion of 5-FU over 24h.

**Table I** Patient characteristics

| | *No. of patients* |
|---|---|
| Sex | |
| Male | 7 |
| Female | 10 |
| Age | |
| Range | 21–75 |
| Median | 55 |
| Prior treatment | |
| None | 1 |
| Surgery only | 10 |
| Chemotherapy only | 1 |
| Surgery + chemotherapy | 5 |
| Tumour type | |
| Colorectal | 10 |
| Ovarian | 4 |
| Gastric | 3 |

*Pharmacokinetic studies*

Pharmacokinetic studies were performed in six patients during the first week of chemotherapy, when multiple samples were withdrawn simultaneously from the i.p. catheter and an indwelling intravenous catheter at the following intervals, timed from the start of the dialysate exchange: 0, 2, 4, 6, 12 and 24 h. The samples were held on ice, centrifuged and the supernatant stored at −20°C until analysed by a sensitive and specific GC–MS assay in our laboratory (Bates *et al*., 1991).

### Results

*Toxicity profile*

The toxicity profile is summarised in Table II. The most consistent problem that interrupted therapy was infective peritonitis (one episode every 12 catheter–weeks). *Aspergillus* and *Pseudomonas* were isolated from two cases and the diagnosis of infective peritonitis was supported in two other patients with the diagnostic triad of severe abdominal pain, elevated peritoneal white cell count ($>200 \times 10^9$ $l^{-1}$) and cloudy peritoneal effluent. Potential causes of the high peritonitis rates included a delivery system that afforded inadequate microbiological protection, a steep learning curve on behalf of patients and impairment of the serosal barrier to infection (we documented relative i.p. hypocomplementaemia in our patients: $C_3$, 0.11 g $l^{-1}$; $C_4$ 0.02 g $l^{-1}$; normal ranges: $C_3$ 0.75 1.75 g $l^{-1}$; $C_4$, 0.14–0.54 g $l^{-1}$). No significant 5-FU-related systemic toxicity was seen at 200 mg $m^{-2}$ per day, therefore the dose was escalated to 300 mg $m^{-2}$ per day. After treating 14 patients, we redesigned the dialysis delivery set, dispensing with the T-set and using one connection point only, protected with a Povidone/iodine shield. Since this change, there have been no further episodes of peritonitis (26 catheter–weeks). Although there were no further episodes of infective peritonitis, it was noted that patients treated for more than 6 weeks at 300 mg $m^{-2}$ suffered significant abdominal pain (WHO grades 2 and 3) and tenderness without elevated peritoneal white cell counts, which was consistent with moderate chemical peritonitis. This was reduced somewhat by oral analgesics, but it was not considered likely that the dose of 5-FU could be

**Table II** Toxicity in 5-FU-treated patients

| *Dose level (mg $m^{-2}$)* | *Number of patients entered* | *Total number of catheter weeks* | *WHO toxicity codes* Abdominal pain 1 | 2 | 3 | 4 | Infective peritonitis 1 | 2 | 3 | 4 | Lethargy 1 | 2 | 3 | 4 | Anorexia 1 | 2 | 3 | 4 | Constipation 1 | 2 | 3 | 4 | Nausea and vomiting 1 | 2 | 3 | 4 |
|---|---|---|---|---|---|---|---|---|---|---|---|---|---|---|---|---|---|---|---|---|---|---|---|---|---|---|
| 200 | 5 | 31 | 1(4) | | 2(7) | | | | 2(2) | | | 1(1) | | | | | | | | | | | | 1(1) | | |
| 300 | 7 | 75 | | 2(6) | 2(4) | | | | 2(3)[a] | | | 1(1) | | | 1(1) | 1(2) | | | | 4(12) | | | | 2(2) | | |

Reported as worst related toxicity per patient at each dose level – the number of patient–weeks experienced is shown in brackets. Five patients with catheter *in situ* did not commence chemotherapy because of catheter-related problems. Diarrhoea (grade 2) was seen in one patient treated with 200 mg $m^{-2}$ 5-FU. If there was no toxicity (WHO grade 0), this is not recorded in the table. [a]No organism was grown from the peritoneal fluid of these patients.

escalated further without a reduction in the duration of therapy.

*Duration of treatment*

Five patients failed to reach the chemotherapy stage–two patients had inadequate distribution of intraperitoneal fluid, one patient had an exit site infection, one patient died post-operatively as a result of a pulmonary embolism and one patient had a bowel obstruction. Twelve patients had chemotherapy, five at 200 mg $m^{-2}$ and seven at 300 mg $m^{-2}$. Individual treatment duration of the 12 patients who received chemotherapy is shown in Table III.

*Pharmacokinetic studies*

Pharmacokinetic studies revealed that it was possible to generate average i.p. 5-FU concentrations of 30 000 ng $ml^{-1}$ (range 23 000–35 000 ng $ml^{-1}$) and corresponding systemic venous levels of 6 ng $ml^{-1}$ (range 1.9–13.7 ng $ml^{-1}$) for a dose of 200 mg $m^{-2}$, and mean i.p. 5FU concentrations of 47 500 ng $ml^{-1}$ (range 29 000–72 000 ng $ml^{-1}$) and associated mean venous levels of 30 ng $ml^{-1}$ (range 11–55 ng $ml^{-1}$) for a dose of 300 mg $m^{-2}$ (Figure 2).

The regional advantage accrued from i.p. infusional 5-FU is expressed as a ratio of i.p./plasma concentrations (mean 6500, range 536–16724) (Table IV). It was not possible to determine a terminal half-life for 5-FU as plasma concentrations were relatively close to the limit of detection and the brief terminal half-life of the drug. There was no clear evidence of circadian variation in plasma 5-FU concentrations; however, this could relate to the number of samples taken.

**Table III** Treatment outcome

| *Weeks of treatment* | *Number of patients discontinued treatment – primary disease* | *Reason off-study* |
|---|---|---|
| 1 | 1–gastric | Disease progression |
| | 1–ovarian | Catheter blockage |
| 2 | 1–colorectal | Absorbing fluid |
| 3 | 1–colorectal | Increasingly poor distribution |
| 4 | 1–ovarian | Peritonitis |
| 5 | 1–ovarian | Disease progression |
| 6 | 1–colorectal | Disease progression |
| | 1–gastric | Peritonitis |
| 7 | | |
| 8 | 1–colorectal | Peritonitis |
| 9 | 1–colorectal | Weight loss |
| 10 | 1–colorectal | Weight loss |
| 11 | | |
| 12 | 1–colorectal | Completed study |

*Follow-up*

Of those 12 patients who received treatment, eight have died (median 10 weeks after end of treatment). Four patients completed more than 8 weeks of treatment, one patient completing 12 weeks. As shown, disease progression and peritonitis were the main reasons for patients being taken off-study early. Of the four patients who had over 8 weeks of treatment, three are alive and well 10–20 months after treatment completion. One such patient had multiple peritoneal metastases at time of insertion of the i.p. catheter.

### Discussion

It would appear that prolonged intraperitoneal infusion of 5-FU, 300 mg $m^{-2}$ per day, is tolerable and generates the sort of pharmacokinetic profile that will take full advantage of the steep dose–response curve for 5-FU, namely very high concentrations within the peritoneal cavity covering peritoneal dissemination of disease and local recurrence; approximately 70% clearance via the portal venous circulation (Speyer *et al.*, 1981), dealing with micrometastases to the liver, with the remainder of the drug being cleared through retroperitoneal lymphatics; and mean plasma levels of 5-FU similar to those described for venous infusions of 5-FU (Harris *et al.*, 1990), providing cover for extrahepatic micrometastases.

Previous, small, randomised studies of adjuvant i.p. 5-FU in colorectal cancer have used intermittent high-dose regimens (dose intensity = 2.3 g $m^{-2}$ per week) that do not make best use of the cell cycle-specific cytotoxic properties of 5-FU (Cunliffe and Sugarbaker, 1989). There is clinical evidence suggesting that prolonged intravenous infusion of 5-FU results in superior response rates in advanced colorectal cancer, compared with intermittent bolus therapy, lending clinical weight to the pharmacological rationale for continuous exposure to 5-FU (Lokich *et al.*, 1989).

One other pharmacological benefit that 5-FU enjoys relative to many other antineoplastic drugs that have been administered via the peritoneal route is its capacity to diffuse relatively homogeneously through tumour tissue (Erlanson *et al.*, 1992). It has been shown that other cytotoxic drugs, such as doxorubicin (Kerr and Kaye, 1987), vinblastine (Nederman *et al.*, 1981) and methotrexate (West *et al.*, 1980), are quite limited in their capacity to diffuse down a concentration gradient into multicellular tumour spheroids *in vitro* or peritoneal tumour nodules *in vivo*. This has been considered to be one of the dominant reasons limiting the clinical

**Figure 2** 5-FU levels in peritoneum and plasma.

**Table IV** Pharmacokinetic results

| Patient ID | Total i.p. 5-FU dose delivered (mg) | Peritoneal AUC ($mg\,l^{-1}\,h$) | Peritoneal Mean conc. ($mg\,l^{-1}$) | Plasma AUC ($mg\,l^{-1}\,h$) | Plasma Mean conc. ($mg\,l^{-1}$) | Regional advantage Peritoneal/Plasma ratio AUC | Regional advantage Peritoneal/Plasma ratio Mean conc. |
|---|---|---|---|---|---|---|---|
| | 200 mg m $^{-2}$ dose | | | | | | |
| 1 | 304 | 1010 | 30.5 | 0.379 | 0.0137 | 2665 | 2223 |
| 2 | 416 | 765 | 23.1 | 0.129 | 0.0019 | 5933 | 12168 |
| 3 | 300 | 976 | 35.1 | 0.145 | 0.0021 | 6730 | 16724 |
| | 300 mg m $^{-2}$ dose | | | | | | |
| 4 | 600 | – | 29.2 | – | 0.055 | – | 536 |
| 5 | 534 | 2003 | 72.1 | 0.58 | 0.022 | 3453 | 3277 |
| 6 | 496 | 969 | 41.2 | 0.379 | 0.011 | 2557 | 3746 |

AUC, area under the concentration–time curve. conc., concentration.

development of i.p. chemotherapy, and lack of tumour penetration could contribute significantly to relative drug resistance (Kerr and Los, 1993).

In the same way that prolonged i.v. infusions of 5-FU did not become widely established until technical innovation resulted in increased pump reliability, i.p. chemotherapy has suffered from a lack of research into improved drug delivery systems. During this study, the chemotherapy-giving set was successfully redesigned to reduce the possibility of bacterial contamination and hence peritonitis. The peritoneal dialysate Icodextrin 7.5% is a macromolecular starch-based polymer similar in structural to glycogen, i.e. non-toxic and broken down in the body by natural carbohydrases. It was developed to increase the peritoneal dwell time of the dialysis fluid, improve control of toxicity and fluid balance and perhaps reduce the possibility of the peritoneal diabetes characteristic of prolonged continuous peritoneal dialysis with conventional low molecular weight dialysates for use in patients with renal failure. The physicochemical properties of Icodextrin greatly simplify drug delivery and make i.p. therapy potentially much more widely applicable.

There is a renewal of interest in i.p. chemotherapy as a result of the recent publication of the positive results of the randomised SWOG/GOG/ECOG study, favouring i.p. over i.v. chemotherapy (Alberts *et al.*, 1995). It was demonstrated that a subgroup of ovarian cancer patients with small volume disease (stage III) showed increased survival by a median of 10 months after i.p. cisplatin compared with i.v. cisplatin (with all patients receiving i.v. cyclophosphamide). Furthermore, a number of experimental and clinical studies have indicated that i.p. therapy in patients with large bulky disease will not improve i.v. treatment in spite of the fact that virtually all agents demonstrated a pharmacological advantage when administered to the peritoneal cavity. The reason for this is that cytostatic drugs penetrate poorly into tumours, resulting in higher drug levels only in the outer cell layers of peritoneal tumours. Consequently, a few cell layers with high drug concentrations will not eradicate large tumour masses. Therefore, patients most likely to benefit from the i.p. therapy are those with a small tumour volume at the start of treatment. In addition, the better the 'penetration pattern' or diffusibility of the cytotoxic agent, the greater the likelihood of benefits. Icodextrin provides a greater variety of i.p. drug administration schedules, particularly for cycle-specific drugs, therefore we plan to draw these different strands together and conduct a pilot study using the schedule described in this report for i.p. chemotherapy in an adjuvant setting, following resection of primary bowel cancer.

**Acknowledgements**

The authors would like to thank Sue Denton, Projects Administrator, Baxter R&D Europe, Nivelles, Belgium, for all her help with this study and to acknowledge the financial support of the Cancer Research Campaign.

### References

ALBERTS DS, LIU PY, HANNIGAN EV, O'TOOLE R, WILLIAMS SD, YOUNG J ON BEHALF OF THE COMBINED GYNAECOLOGICAL STUDY GROUP. (1995). Phase II study of intraperitoneal cisplatin/intravenous cyclophosphamide vs intravenous cisplatin/intravenous cyclophosphamide in patients with optimal disease stage III ovarian cancer: a SWOG GOG ECOG intergroup study (INT 0051). *Proc. Am. Soc. Clin. Oncol.*, **14**, 273.

BATES CD, WATSON DG, WILLMOTT N, LOGAN H AND GOLDBERG J. (1991). The analysis of 5-FU in human plasma by gas chromatography - negative ion chemical ionisation mass spectrometry (GG-NICIMS) with stable isotype dilution. *J. Pharm. Biomed. Anal.*, **9**, 19–21.

CUNLIFFE WJ AND SUGARBAKER PH. (1989). Gastrointestinal malignancy: rationale for adjuvant therapy using early postoperative intraperitoneal chemotherapy (EPIC). *Br. J. Surg.*, **76**, 1082–1090.

ERLANSON M, DANIEL-SZOLGAY E AND CARLSSON. J. (1992). Relations between penetration, binding and average concentration of cytostatic drugs in human tumour spheroids. *Cancer Chemother. Pharmacol.*, **29**, 343–353.

GRAY R, JAMES R, MOSSMAN J AND STENNING S. (1991). AXIS – A suitable case for treatment. *Br. J. Cancer*, **63**, 8441–8445.

HARRIS BE, SONG R, SOONG SJ AND DIASIO RB. (1990). Relationships between dihydropyrimidine dehydrogenase activity and plasma 5-FU levels with evidence for circadian variation of enzyme activity and plasma drug levels in cancer patients receiving 5-FU by protracted continuous infusion. *Cancer Res.*, **50**, 197–201.

LOKICH JJ, AHLGREN JD, GULLO JJ, PHILIPS JA AND FRYER JG. (1989). A prospective randomised comparison of continuous infusion fluorouracil with a conventional bolus schedule in metastatic colorectal cancer. *J. Clin. Oncol.*, **7**, 425–432.

KERR DJ AND KAYE SB. (1987). Aspects of cytotoxic drug penetration with particular reference to anthracyclines. *Cancer Chemother. Pharmacol.*, **19**, 1–5.

KERR DJ AND LOS G. (1993). Pharmacokinetic principles of locoregional chemotherapy. *Cancer Surv.*, **17**, 105–122.

MCARDLE CS, KERR DJ, O'GORMAN P, WOTHERSPOON HA, WARREN H, WATSON D, VINCKE BJ, DOBBIE JW AND EL EINI DID. (1994). Pharmacokinetic study of 5-FU in a novel dialysate solution: a long term IP treatment approach for colorectal cancer. *Br. J. Cancer*, **70**, 762–766.

NEDERMAN T, CARLSSON J AND MALMQUIST M. (1981). Penetration of substances into tumour tissue. A methodological study on cellular spheroids. *In Vitro*, **17**, 290 298.

SPEYER JL, SUGARBAKER PH, COLLINS JM, DEDRICK RL, KLECKER RW AND MYERS CE. (1981). Portal levels and hepatic clearance of 5-FU after IP administration in tumours. *Cancer Res.*, **41**, 1916–1922.

WEST GW, WEICHSELBAUM R AND LITTLE JB. (1980). Limited penetration of methotrexate into human osteosarcoma spheroids as a proposed model for solid tumour resistance to adjuvant chemotherapy. *Cancer Res.*, **40**, 3665–3668.